# Benzo[*a*]pyrene-Induced Genotoxicity in Rats Is Affected by Co-Exposure to Sudan I by Altering the Expression of Biotransformation Enzymes

**DOI:** 10.3390/ijms22158062

**Published:** 2021-07-28

**Authors:** Helena Dračínská, Radek Indra, Sandra Jelínková, Věra Černá, Volker Manfred Arlt, Marie Stiborová

**Affiliations:** 1 Department of Biochemistry, Faculty of Science, Charles University, Hlavova 8, 12843 Prague, Czech Republic; radek.indra@natur.cuni.cz (R.I.); sandra.jelinkova@lf1.cuni.cz (S.J.); vera.cerna@natur.cuni.cz (V.Č.); 2 Toxicology Department, GAB Consulting GmbH, 69126 Heidelberg, Germany; volker.arlt@gabconsulting.de

**Keywords:** benzo[*a*]pyrene, Sudan I, genotoxicity, DNA-adducts, cytochromes P450 1A1 and 1A2 and 1B1, microsomal epoxide hydrolase

## Abstract

The environmental pollutant benzo[*a*]pyrene (BaP) is a human carcinogen that reacts with DNA after metabolic activation catalysed by cytochromes P450 (CYP) 1A1 and 1B1 together with microsomal epoxide hydrolase. The azo dye Sudan I is a potent inducer of CYP1A1/2. Here, Wistar rats were either treated with single doses of BaP (150 mg/kg bw) or Sudan I (50 mg/kg bw) alone or with both compounds in combination to explore BaP-derived DNA adduct formation *in vivo*. Using ^32^P-postlabelling, DNA adducts generated by BaP-7,8-dihydrodiol-9,10-epoxide were found in livers of rats treated with BaP alone or co-exposed to Sudan I. During co-exposure to Sudan I prior to BaP treatment, BaP-DNA adduct levels increased 2.1-fold in comparison to BaP treatment alone. Similarly, hepatic microsomes isolated from rats exposed to Sudan I prior to BaP treatment were also the most effective in generating DNA adducts *in vitro* with the activated metabolites BaP-7,8-dihydrodiol or BaP-9-ol as intermediates. DNA adduct formation correlated with changes in the expression and/or enzyme activities of CYP1A1, 1A2 and 1B1 in hepatic microsomes. Thus, BaP genotoxicity in rats *in vivo* appears to be related to the enhanced expression and/or activity of hepatic CYP1A1/2 and 1B1 caused by exposure of rats to the studied compounds. Our results indicate that the industrially employed azo dye Sudan I potentiates the genotoxicity of the human carcinogen BaP, and exposure to both substances at the same time seems to be hazardous to humans.

## 1. Introduction

Numerous xenobiotics play an important role in the development of many cancers. The polycyclic aromatic hydrocarbon benzo[*a*]pyrene (BaP) belongs to the most extended proven human carcinogens in the environment [1]. BaP is formed by incomplete combustion or pyrolysis of organic matter and it can be found in diesel exhaust, tobacco smoke and food either processed at high temperatures or grown in contaminated soils [2]. Thus, human exposure to BaP is almost ubiquitous and permanent due to diet and environmental sources.

**Figure 1:**
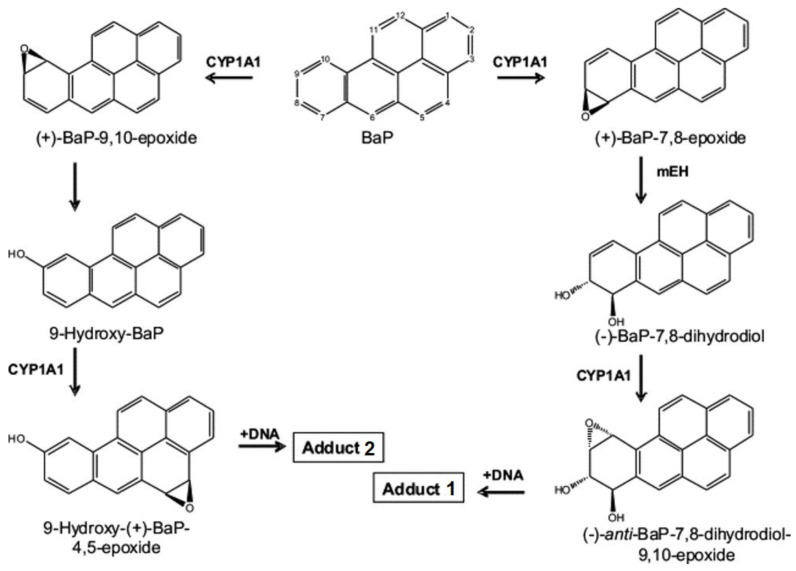
Scheme of BaP biotransformation pathways and DNA adduct formation catalysed by CYP1A1 and mEH (taken and edited from [6]).

To exert its genotoxic effect, BaP requires metabolic activation [3]. Cytochrome P450 (CYP) 1A1 in conjunction with microsomal epoxide hydrolase (mEH) are considered the most important enzymes for BaP bioactivation to highly reactive derivatives capable of binding to DNA [4]. Activated BaP metabolites forming the major DNA adducts are BaP-7,8-dihydrodiol-9,10-epoxide (BPDE) and 9-hydroxy-BaP-4,5-epoxide (see the scheme of BaP-derived adduct formation in Figure 1) [5,6]. The oxidation of BaP is also mediated by CYP1B1, but with lower efficiency than that of CYP1A1 [7,8,9], and to a very small extent by some other CYP isoforms (e.g., CYP1A2, CYP2C) [9,10].

The suspected human carcinogen Sudan I (1-phenylazo-2-hydroxynaphthol or C.I. Solvent Yellow 14) [11] is an azo dye which is widely used to colour materials such as hydrocarbon solvents, oils, fats, waxes, plastics, printing inks and shoe and floor polishes. In the past, Sudan I was also used as an orange food colouring, but it has been prohibited in the European Union and some other countries since 2003 [12], because it causes liver and urinary bladder tumours in rats, mice and rabbits and it is considered a possible human carcinogen [13]. Despite all recommendations, the use of Sudan I as a food colourant is still not completely avoided [14,15,16].

Because modern civilisation is almost constantly exposed to several harmful substances concurrently, one environmental carcinogen might affect the potency of another carcinogen when humans are co-exposed to both. Therefore, using the rat as a mammalian model, we investigated whether and how the genotoxicity of widespread carcinogenic pollutant BaP is affected *in vivo* when co-exposed with another potential carcinogen, Sudan I. Exposure to BaP is unavoidable and comes mainly from food but also from polluted air, and in case of smokers from cigarettes. Thus, co-exposure to both compounds may occur in workers in industries where Sudan I dye is used, or in consumers, mainly in non-EU markets, where curry and chili spices or tomato sauces are still sometimes coloured and adulterated with Sudan I [16,17].

For the purpose of this study, rats were either exposed to BaP or Sudan I alone or to both compounds in combinations. Different exposure scenarios were considered: (*i*) BaP treatment alone; (*ii*) Sudan I treatment alone; (*iii*) Sudan I exposure prior to BaP treatment; and (*iv*) BaP exposure prior to Sudan I treatment. As a measure of genotoxicity, DNA adduct formation was determined by ^32^P-postlabelling and BaP-derived DNA adduct levels were compared to the expression and/or activity of major biotransformation enzymes crucial for metabolic activation of BaP to its genotoxic derivatives.

Sudan I and BaP are both predominantly activated by CYP1A1 [18,19,20], an enzyme that plays a key role in the metabolism of many carcinogens. Furthermore, both compounds are strong inducers of CYP1A1 and other enzymes whose expression is controlled by the aryl hydrocarbon receptor [21,22,23,24,25]. By inducing the expression of their major biotransformation enzyme, CYP1A1, both compounds might affect their own metabolism impacting on the activation and detoxification pathways. This is important to understand for BaP because CYP1A1 has been shown in most studies to be responsible for BaP bioactivation *in vitro* [3,4,5,26,27], whereas studies in knock-out animal models indicated the importance of CYP1A1 for BaP detoxification *in vivo* [20,28,29,30]. Therefore, one of the aims of this study was to resolve this discrepancy in the role of CYP1A1 in BaP genotoxicity. We investigated whether increased levels of CYP1A1, induced by Sudan I administrated to rats one day prior to BaP treatment, lead to any changes in BaP-derived DNA adduct levels in rat liver *in vivo*.

## 2. Results

### 2.1. Formation of BaP-Derived DNA Adducts In Vivo in Rats Exposed to BaP Alone or in Combination with Sudan I

**Figure 2:**
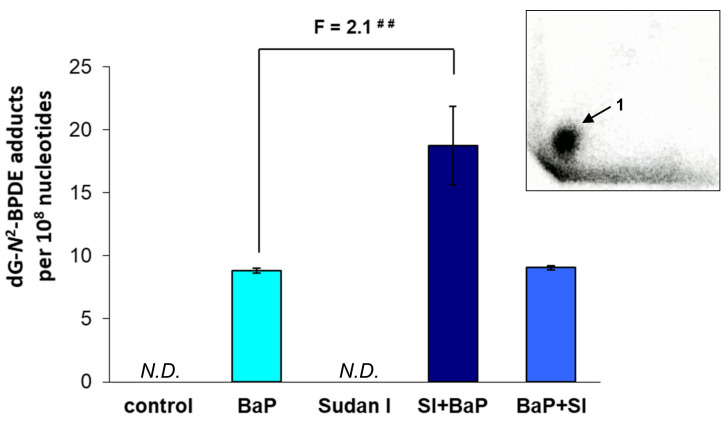
Quantitative TLC-^32^P-postlabelling analysis of DNA adducts in livers of control (untreated) rats and rats treated with BaP alone, Sudan I alone, Sudan I before BaP treatment (SI + BaP) or Sudan I after BaP treatment (BaP + SI). Number above columns (“F”) indicates fold change in DNA adduct level in comparison to animals treated with BaP alone. The values represent mean ± S.D. of three liver samples/group; each DNA sample was determined by two ^32^P-postlabelling analyses. Statistical comparison was performed by t-test analysis; ^##^ *p* < 0.01—results significantly different from animals treated with BaP alone. *N.D.*—not detected. Insert: Representative autoradiogram showing the dG-*N*^2^-BPDE adduct (assigned adduct 1) formed in livers of rats treated with BaP; the origin on the TLC plate, at the bottom left-hand corner, was cut off before imaging.

The nuclease P1 enrichment version of the ^32^P-postlabelling assay was employed to determine the formation of covalent DNA adducts (i.e., 10-(deoxyguanosin-*N*^2^-yl)-7,8,9-trihydroxy-7,8,9,10-tetrahydro-BaP [dG-*N*^2^-BDPE]) in rat livers of all treatment groups. No BaP-derived adducts were found in the livers of control (untreated) rats and rats treated with Sudan I alone (data not shown). One major DNA adduct spot (insert in Figure 2) was found in the livers of all rats exposed to BaP either alone or in combination with Sudan I, which was applied one day before or one day after BaP treatment. This adduct spot (assigned adduct 1) was previously identified as dG-*N*^2^-BPDE, which is formed by BaP-7,8-dihydrodiol-9,10-epoxide (BPDE) bound to guanine in DNA [29].

The levels of dG-*N*^2^-BPDE formed *in vivo* in livers of rats treated with BaP either alone or in combination with Sudan I differed among the treatment groups (Figure 2). They increased significantly in the case of the exposure of rats to Sudan I one day before BaP treatment, being ~2 times higher than in rats treated with BaP alone. In contrast, application of Sudan I one day after BaP treatment did not alter BaP-derived DNA adduct formation compared to rats treated with BaP alone.

### 2.2. Formation of BaP-Derived DNA Adducts after Incubation of BaP with Rat Hepatic Microsomes In Vitro

**Figure 3:**
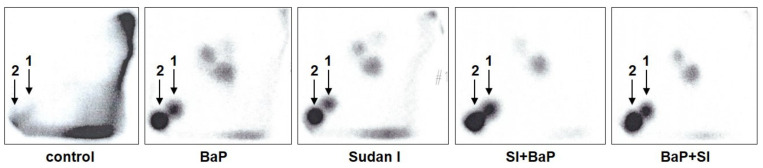
Autoradiographic profiles of DNA adducts formed in incubations of calf thymus DNA with BaP, NADPH (cofactor) and hepatic microsomes isolated from livers of control (untreated) rats and rats exposed to BaP alone, Sudan I alone, Sudan I before BaP treatment (SI + BaP) or Sudan I after BaP treatment (BaP + SI). Adduct 1—dG-*N*^2^-BPDE; adduct 2—hydroxy-BaP-4,5-epoxide-derived guanine adduct. The origin on the TLC plate, at the bottom left-hand corners, was cut off before imaging.

Hepatic microsomes isolated from rats of each treatment group [control (untreated), BaP alone, Sudan I alone, Sudan I before BaP treatment or Sudan I after BaP treatment] were incubated with BaP and NADPH, as a cofactor of the CYP-mediated BaP oxidation, in the presence of calf thymus DNA. BaP-derived DNA adducts formed during the incubations were analysed by ^32^P-postlabelling. As shown in Figure 3, two major adduct spots (assigned adduct 1 and 2) were formed in BaP incubation with hepatic microsomes isolated from rats of all treatment groups. As found *in vivo* (compare Figure 2), adduct 1 was again identified as dG-*N*^2^-BPDE [29], whereas adduct 2, not detected *in vivo*, corresponded to deoxyguanosine adducts derived from the reaction with 9-hydroxy-BaP-4,5-epoxide [31] (see scheme in Figure 1). In addition, two or three minor adduct spots were also visible after autoradiography (Figure 3). It has been suggested previously that they also can be BaP-derived DNA adducts [32,33]. Indeed, these minor adducts were not found in control incubations carried out without BaP. As the origin of these adducts is currently unknown, their levels were not quantified in the present study.

**Figure 4:**
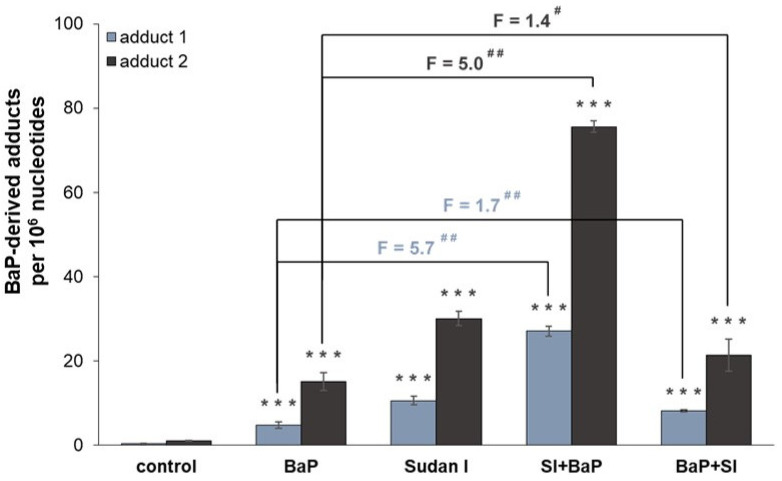
DNA adduct formation by BaP, measured by TLC-^32^P-postlabelling, after activation with hepatic microsomes isolated from livers of control (untreated) rats and rats exposed to BaP alone, Sudan I alone, Sudan I before BaP treatment (SI + BaP) or Sudan I after BaP treatment (BaP + SI). The levels of BaP-derived adducts were related to 1 nmol of total microsomal CYP in the incubation. Numbers above columns (“F”) indicate fold changes in DNA adduct level in comparison to incubations with microsomes from animals treated with BaP alone. The values represent mean ± S.D. of three independent incubations. Statistical comparison was performed by *t*-test analysis: ^#^ *p* < 0.05, ^##^ *p* < 0.01—results significantly different from incubations with microsomes of rats treated with BaP alone; *** *p* < 0.001—significantly different from incubations with microsomes of control rats. Adduct 1—dG-*N*^2^-BPDE; adduct 2—9-hydroxy-BaP-4,5-epoxide-derived guanine adduct.

Total BaP-DNA adduct levels were increased by one order of magnitude in case of hepatic microsomes isolated from rats pre-treated with either BaP alone, Sudan I alone or both compounds in combinations compared to those catalysed by hepatic microsomes of control (untreated) rats (Figure 4). BaP-DNA adduct levels were the highest in incubations containing BaP and hepatic microsomes of rats treated with Sudan I one day before BaP treatment. The levels of dG-*N*^2^-BPDE and 9-hydroxy-BaP-4,5-epoxide-derived adducts formed *in vitro* using hepatic microsomes of these rats were 70–80-fold higher in comparison to the microsomes from control (untreated) rats and approximately 5–6-fold higher in comparison to the microsomes from rats treated only with BaP. These results are in concordance with those found in rat livers *in vivo* (r = 0.997, *p* < 0.05). There, the highest dG-*N*^2^-BPDE levels were also formed in livers of rats exposed to Sudan I one day before BaP treatment (Figure 2). Further, not only combined exposure of rats to Sudan I one day before BaP treatment but also exposure of rats to Sudan I one day after BaP treatment increased the efficiency of microsomes to catalyse BaP-derived DNA adduct formation *in vitro* compared to hepatic microsomes from rats treated with BaP alone, although to a much lower extent (Figure 4).

In order to better understand the observed changes in BaP-derived DNA adduct formation *in vivo* and *in vitro*, the role of BaP biotransformation enzymes including CYP1A1, 1A2 and 1B1 [3,34,35] was further investigated. The effect of exposure to BaP alone, Sudan I alone or both compounds in combination on the expression and activity of these enzymes was examined in rat liver. Another approach to assessing changes in the expression of studied CYP isoforms was the determination of their gene expression. Effects on mEH, another enzyme crucial for BaP activation, on the gene and protein expression were also evaluated.

### 2.3. Protein Expression of CYP1A1, CYP1A2 and CYP1B1 in Livers of Rats Exposed to BaP Alone or in Combination with Sudan I

**Figure 5:**
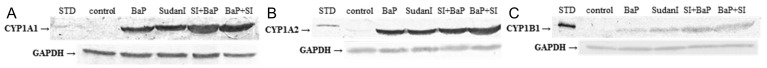
Western blot analysis of CYP1A1 (**A**), 1A2 (**B**) and 1B1 (**C**) in hepatic microsomes isolated from control (untreated) rats and rats exposed to BaP alone, Sudan I alone, Sudan I before BaP treatment (SI + BaP) or Sudan I after BaP treatment (BaP + SI). Glyceraldehyde 3-phosphate dehydrogenase (GAPDH) protein expression was used as a loading control. As a standard (STD) of respective CYP, recombinant rat CYP1A1, rat CYP1A2 or human CYP1B1 in Supersomes™ were used.

The protein levels of CYP1A1, CYP1A2 and CYP1B1 were evaluated by Western blotting using consecutive immunodetection by specific antibodies. In comparison to livers of control (untreated) rats, administration of BaP and Sudan I alone or in combinations led to significant increases in hepatic CYP1A1, 1A2 and 1B1 expression, with greater expression always observed in the case of combined treatment (Figure 5).

For CYP1A1, the most important BaP metabolising enzyme [5,6,9], its expression was hardly detectable in hepatic microsomes of control (untreated) rats indicating that the sensitivity of the primary antibody used was probably too weak to detect very low content of CYP1A1 in rat liver. Nevertheless, CYP1A1 expression was clearly detectable in hepatic microsomes isolated from rats exposed to BaP, Sudan I alone or in combinations (Figure 5A). The scanned blots show that exposure of rats to Sudan I before or after BaP treatment slightly increased CYP1A1 expression in comparison to the application of BaP or Sudan I alone. Similar results were obtained for the highly homologous enzyme of 1A subfamily, CYP1A2 (Figure 5B). However, unlike CYP1A1, CYP1A2 protein expression was clearly detectable also in microsomal samples of control (untreated) rats. Treatment with BaP and Sudan I, either alone or in combinations, enhanced CYP1A2 expression, with a CYP1A1-like increase of CYP1A2 content in hepatic microsomes of rats exposed to BaP in both combinations with Sudan I compared to microsomes of rats treated with BaP or Sudan I alone.

CYP1B1 is another biotransformation enzyme that plays an important role in BaP oxidation [5,7]. Administration of BaP and Sudan I, either alone or in combinations, led to an induction of CYP1B1 expression (Figure 5C). Sudan I exhibited a stronger induction effect than BaP. In comparison to rats treated with BaP or Sudan I alone, combined administration of Sudan I before or after BaP treatment led to a more pronounced induction of protein expression for CYP1B1 than for CYP1A1/2. Overall, exposure of rats to BaP and Sudan I in combinations led to considerably higher protein levels of CYP1A1, CYP1A2 and CYP1B1 than when only exposed to individual compounds.

### 2.4. Enzymatic Activity of CYP1A1, CYP1A2 and CYP1B1 in Livers of Rats Exposed to BaP Alone or in Combination with Sudan I

**Figure 6:**
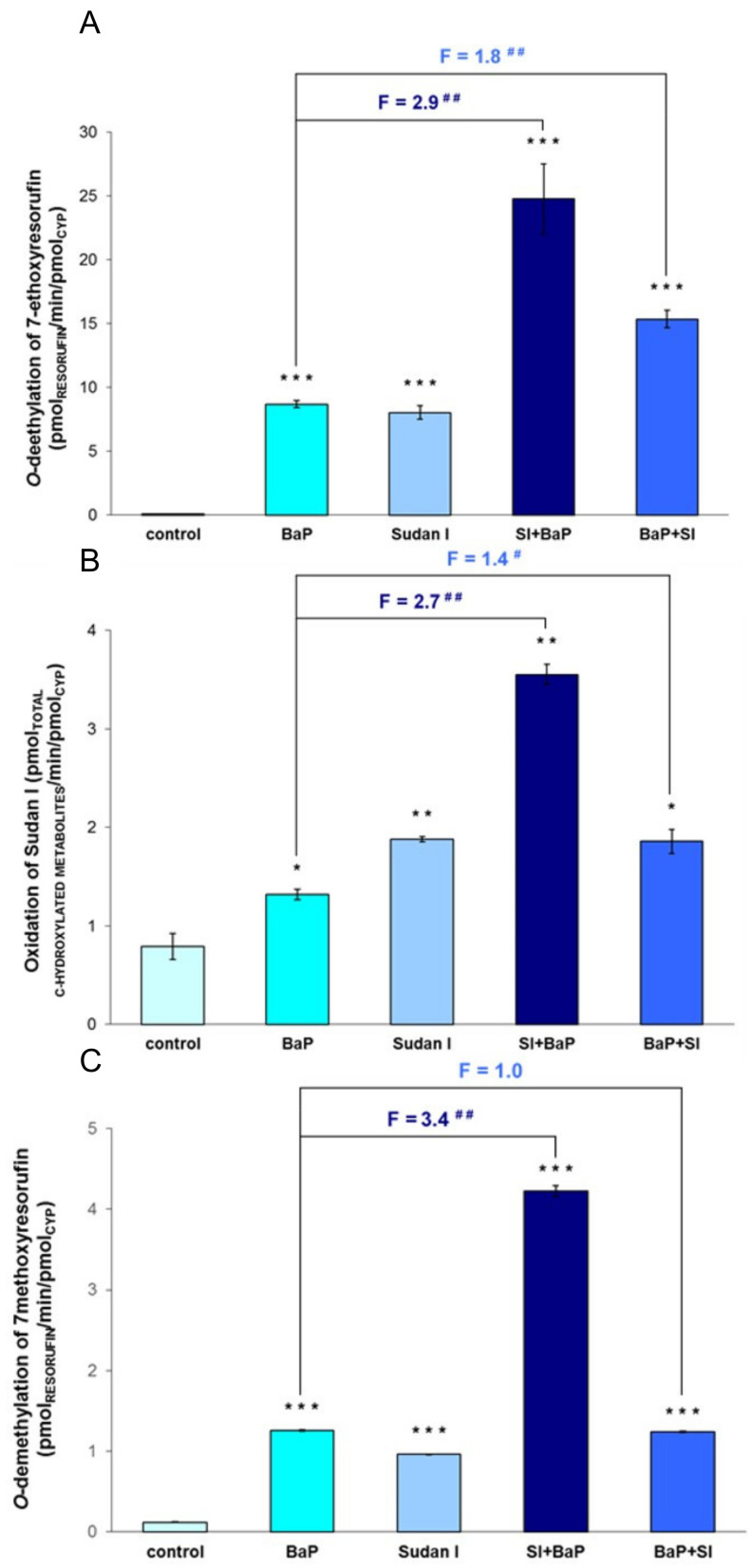
CYP1A1 and 1A2 enzyme activities measured by marker reactions of 7-ethoxyresorufin *O*-deethylation for CYP1A1 (**A**), Sudan I oxidation for CYP1A1 (**B**) and 7-methoxyresorufin *O*-demethylation for CYP1A2 (**C**) in hepatic microsomes isolated from control (untreated) rats and rats exposed to BaP alone, Sudan I alone, Sudan I before BaP treatment (SI + BaP) or Sudan I after BaP treatment (BaP + SI). All values are given as the means ± S.D. of three parallel measurements. Numbers above columns (“F”) indicate fold changes in enzyme activities in comparison to animals treated with BaP alone. Statistical comparison was performed by *t*-test analysis: ^#^ *p* < 0.05, ^##^ *p* < 0.01—results significantly different from rats treated with BaP alone; * *p* < 0.05, ** *p* < 0.01, *** *p* < 0.001—significantly different from control (untreated) rats.

In order to confirm the results obtained by Western blotting, the influence of BaP, Sudan I and their combined applications on CYP enzyme activities were studied in rat livers. 7-Ethoxyresorufine-*O*-deethylation activity (EROD), a marker activity for CYP1A1 [36], was significantly induced by up to two orders of magnitude in hepatic microsomes of rats treated with BaP and/or Sudan I as compared to EROD activity in livers of control (untreated) rats (Figure 6A). Hepatic microsomes of rats exposed to Sudan I one day before BaP treatment exhibited the highest EROD activity, which was increased approximately 3-fold compared to rats treated with BaP alone.

As shown in Figure 6B, similar results were obtained when CYP1A1 enzyme activity was assessed by the oxidation of Sudan I to its C-hydroxylated metabolites [18,37]. Hepatic microsomes of rats exposed to Sudan I before BaP treatment oxidised Sudan I more effectively than hepatic microsomes of rats treated with BaP or Sudan I alone. Here, the sum of total hydroxylated Sudan I metabolites was 2.7 times greater than that formed by hepatic microsomes of rats exposed to BaP alone (Figure 6B). This is in accordance with the increased EROD activity measured in these hepatic microsomes. However, the potency of BaP and Sudan I administered alone or in combinations to induce EROD activity and Sudan I oxidation differed. In comparison to control (untreated) rats, induction of CYP1A1 activity measured by Sudan I oxidation was less pronounced after BaP/Sudan I treatment compared to changes in CYP1A1 activity measured by EROD. It seems that in the case of Sudan I hydroxylation, other dominant CYP isoforms present in rat livers probably contributed to the reaction mediated by control (untreated) microsomes [19]. On the other hand, the fold induction changes observed in rats exposed to Sudan I before or after BaP treatment compared to rats treated with BaP alone were remarkedly similar in both assays measuring CYP1A1 enzyme activity.

7-Methoxyresorufine-*O*-demethylation activity (MROD) was used as a marker reaction for CYP1A2 [36]. CYP1A2 activity was significantly increased in hepatic microsomes of rats treated with BaP alone (10-fold), Sudan I alone (8-fold) or Sudan I one day after BaP treatment (10-fold) relative to control (untreated) rats (Figure 6C). Similar to the results obtained with the EROD assay, the highest increase (35-fold) in MROD activity was measured in hepatic microsomes of rats exposed to Sudan I one day before BaP treatment (Figure 6C).

Interestingly, the measured enzyme activities for EROD, Sudan I oxidation and MROD in hepatic microsomes isolated from rats exposed to Sudan I before BaP treatment were always higher than the sum of respective enzyme activities in hepatic microsomes of rats exposed to a single dose of BaP or Sudan I (Figure 6). Further, in all treatment groups, all determined specific enzyme activities in hepatic microsomes correlated well with the degree of BaP-derived DNA adduct formation catalysed by the same microsomes in vitro (EROD: r = 0.909, *p* < 0.05 for adduct 1 and r = 0.902, *p* < 0.05 for adduct 2; Sudan I oxidation: r = 0.995, *p* < 0.01 for adduct 1 and r = 0.991, *p* < 0.01 for adduct 2; MROD: r = 0.963, *p* < 0.01 for adduct 1 and r = 0.965, *p* < 0.01 for adduct 2).

### 2.5. Gene Expression of CYP1A1, CYP1A2 and CYP1B1 in Livers of Rats Exposed to BaP Alone or in Combination with Sudan I

Quantitative PCR was used to evaluate relative gene expression of *CYP1A1*, *CYP1A2* and *CYP1B1* in rat livers. The transcription of all three genes is controlled by the Ah-receptor [38]. In comparison to control (untreated) rats, exposure to BaP and Sudan I alone or both compounds in combinations led to high mRNA induction of all studied genes (Table 1). The strongest inductive change in *CYP* genes occurred in the expression of *CYP1B1* (up to almost 11,000-fold), followed by *CYP1A1* (up to ~3400-fold), both CYP isoforms with negligible constitutive expression in the liver. The increase in *CYP1A2* gene expression by the test compounds was two orders of magnitude lower (up to 28-fold) than the increase in *CYP1A1* and *1B1* gene expression but still significant. For *CYP1B1*, exposure to Sudan I before and after BaP treatment significantly enhanced the amount of *CYP1B1* mRNA in comparison to rats treated with BaP alone. However, exposure to Sudan I before BaP treatment produced a weaker increase in *CYP1A1/2* mRNA induction than exposure to BaP and Sudan I alone or Sudan I after BaP treatment.

### 2.6. Protein and Gene Expression of mEH in Livers of Rats Exposed to BaP Alone or in Combination with Sudan I

**Figure 7:**
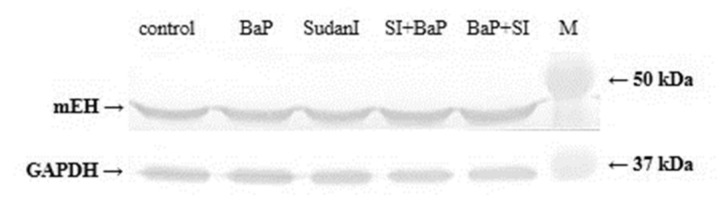
Western blot analysis of mEH in hepatic microsomes isolated from control (untreated) rats and rats exposed to BaP alone, Sudan I alone, Sudan I (SI) before BaP treatment or Sudan I after BaP treatment. GAPDH protein expression was used as a loading control. M—marker of molecular weights.

mEH catalyses biotransformation of BaP-7,8-epoxide to BaP-7,8-dihydrodiol in the reaction pathway leading to the formation of BPDE capable of binding to DNA (i.e., dG-*N*^2^-BPDE) [6]. A slight increase in mEH expression was observed using Western blotting in hepatic microsomes of rats exposed to BaP and Sudan I or both compounds in combinations compared to mEH levels in livers of control (untreated) rats (Figure 7). Increased mEH expression was most prominent in the liver of rats exposed to both test compounds. Similar results were found when mEH gene expression was investigated (Table 2). However, it must be acknowledged that the highest induction potential for Sudan I exposure after BaP treatment was not statistically significant.

### 2.7. Metabolism of BaP by Hepatic Microsomes Isolated from Rats Exposed to BaP Alone or in Combinations with Sudan I

**Figure 8:**
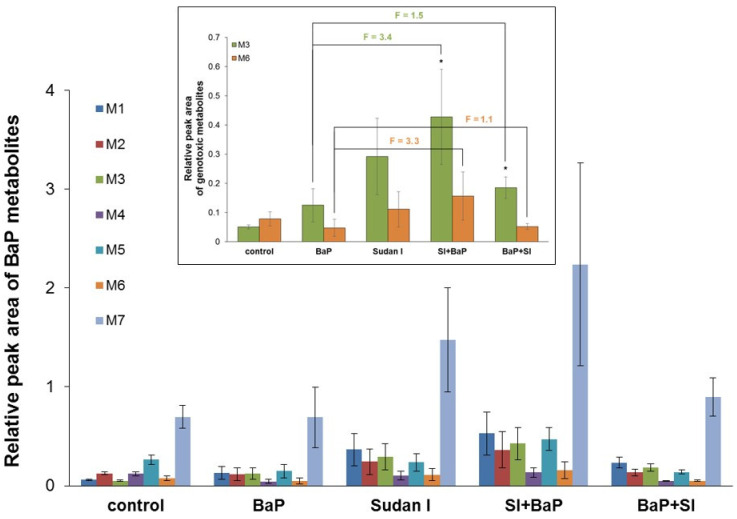
HPLC detection of BaP metabolites from *in vitro* incubations of BaP, NADPH and hepatic microsomal fractions from control (untreated) rats and rats exposed to BaP alone, Sudan I alone, Sudan I before BaP treatment (SI + BaP) or Sudan I after BaP treatment (BaP + SI). Peak areas of BaP metabolites were related to 1 nmol of total microsomal CYP. Values are given as mean ± S.D. from three parallel experiments. M1—BaP-9,10-dihydrodiol, M2—BaP-4,5-dihydrodiol, M3—BaP-7,8-dihydrodiol, M4—BaP-1,6-dione, M5—BaP-3,6-dione, M6—BaP-9-ol, M7—BaP-3-ol. **Insert**: Detailed excerpt from the graph showing results of HPLC detection of genotoxic BaP metabolites M3 (BaP-7,8-dihydrodiol) and M6 (BaP-9-ol). Numbers above columns (“F”) indicate fold changes in relative peak areas in comparison to animals treated with BaP alone. Statistical comparison was performed by *t*-test analysis: no statistically significant difference between rats exposed to BaP alone and BaP in combination with Sudan I; * *p* < 0.05—significantly different from control (untreated) rats.

Seven BaP metabolites were separated by HLPC after BaP incubation with individual hepatic microsomes of rats of all treatment groups in the presence of NADPH as a cofactor of CYP-mediated BaP oxidation: BaP-9,10-dihydrodiol (M1), BaP-4,5-dihydrodiol (M2), BaP-7,8-dihydrodiol (M3), BaP-1,6-dione (M4), BaP-3,6-dione (M5), BaP-9-ol (M6) and BaP-3-ol (M7). No BaP metabolites were formed when NADPH was absent in the reaction mixture. Hepatic microsomes isolated from rats exposed to Sudan I before BaP treatment showed the strongest potency to oxidise BaP, followed by microsomes of rats exposed to Sudan I alone (Figure 8).

The metabolites BaP-7,8-dihydrodiol (M3) and BaP-9-ol (M6) are considered to be precursors of reactive BaP derivatives able to bind covalently to the DNA (Figure 1), forming BaP-derived DNA adducts (i.e., adduct 1 and 2 as shown in Figure 2 and Figure 3) [39]. In the present study, formation of the genotoxic metabolites BaP-7,8-dihydrodiol (M3) and BaP-9-ol (M6) was mainly catalysed by hepatic microsomes of rats exposed to Sudan I before BaP treatment (insert in Figure 8). The ability of individual hepatic microsomes of each treatment group to form BaP-7,8-dihydrodiol (M3) corresponded to CYP1A1 activity (Figure 5) in respective microsomes (r = 0.957, *p* < 0.05) and levels of dG-*N*^2^-BPDE formed *in vitro* (Figure 3) by these microsomes (r = 0.961, *p* < 0.01).

## 3. Discussion

In the present study, we investigated the genotoxic properties of ubiquitous carcinogen BaP when BaP acted together with another potential carcinogen Sudan I [19], which, like BaP [22,25,26,40], is a potent inducer of CYP1A1 [23,24], a key enzyme catalysing BaP activation to genotoxic metabolites [3,6,41]. Both *in vivo* and *in vitro* experimental approaches were chosen in order to obtain the most evident results. Wistar rats were exposed to a single dose of either BaP or Sudan I alone, or rats were exposed to the combinations of both compounds: one group of rats receiving Sudan I one day before BaP treatment and another group one day after BaP treatment. Treatment conditions of both compounds in single doses were based on previous studies in which similar doses exhibited carcinogenic properties [12,24,26,42,43]. Relatively high doses were administrated to rats to manifest the studied phenomena even after a single administration. Thus, dose levels have been selected to study mechanisms underlying carcinogenesis and are not based on dietary exposure levels. People’s daily dietary intake of BaP varies between units and hundreds of ng per person per day [44,45,46] but lasts for a lifetime. Moreover, for smokers, approximately 2–24 ng of BaP were found in one cigarette [47]. Sudan I intake depends on the consumption of contaminated chili/turmeric/curry/tomato products containing this dye, which are present in amounts corresponding to units up to thousands of mg per kg [12,15,16].

In addition to evaluating BaP-derived DNA adduct formation *in vivo* in the liver of rats exposed to BaP, several other approaches were employed in order to better understand possible effects on mechanisms underlying BaP genotoxicity: (*i*) ability of microsomes isolated from liver of treated rats to mediate BaP metabolism *in vitro*; (*ii*) ability of hepatic microsomes to catalyse the formation of BaP-derived DNA-adducts *in vitro* in the presence of NADPH as a co-factor of CYP-mediated bioactivation; and (*iii*) expression of hepatic CYP isoforms involved in BaP biotransformation, namely CYP1A1, 1A2 and 1B1 [48], at the gene and protein levels as well as their enzyme activities.

Sudan I has been shown to form DNA adducts *in vivo* [49], however because they are difficult to detect by ^32^P-postlabelling in the livers of rats exposed to this azo dye, Sudan I-induced DNA adducts were not determined in the present study as the focus of investigation was on BaP-induced genotoxicity.

One major adduct identified as dG-*N*^2^-BPDE, which results from the binding of the activated metabolite BaP-7,8-dihydrodiol-9,10-epoxide to guanine bases in DNA [29,41,50,51], was found in the livers of all BaP-exposed rats (insert in Figure 2). Furthermore, this adduct was also identified as a major DNA adduct in mouse [52,53] or in human liver cells [54]. The potential of BaP to generate DNA adducts was also observed in fish [55]. However, the level of DNA adducts likely differ between species as BaP metabolism differs significantly between species [56]. Interestingly, adduct levels were similar in the livers of rats exposed only to BaP one day before sacrifice and rats exposed to Sudan I one day after BaP treatment, meaning that rats had been exposed to BaP two days prior to sacrifice. Despite the time difference in the administration of BaP, these results indicate that BaP-derived DNA adducts were not readily eliminated from the damaged DNA two days after application. Similar results have been reported by others previously [33,57]. Nevertheless, the highest levels of dG-*N*^2^-BPDE were found in the livers of rats exposed to Sudan I one day prior to BaP treatment indicating that pre-treatment with Sudan I prior to BaP exposure leads to increased formation of activated BaP metabolites which are capable of binding to DNA. A likely reason to explain this observation is the overexpression of CYP family 1 isoenzymes, which are induced by both Sudan I and BaP [23,24,58], as has also been shown in the current experiments.

The results obtained from the detection of BaP-DNA adducts *in vivo* were consistent with findings for BaP-derived DNA adduct formation *in vitro* after metabolic activation of BaP by hepatic microsomal systems of individual rat groups in the presence of NADPH (Figure 4). These results suggest a crucial role of microsomal CYP enzymes that use NADPH as a catalytic cofactor in BaP activation. Microsomes isolated from the liver of rats exposed first to Sudan I and then to BaP were most effective in converting BaP to intermediates capable of forming DNA adducts, reflecting the situation *in vivo*. In contrast to the *in vivo* situation, two DNA adducts were formed in microsomal incubations *in vitro*. In addition to dG-*N*^2^-BPDE, a major deoxyguanosine adduct derived from 9-hydroxy-BaP-4,5-epoxide was also detected. The same phenomenon of differences between the *in vivo* and *in vitro* BaP-derived DNA adduct pattern obtained by ^32^P-postlabelling analysis has already been observed before [59]. This might be due to the presence of only membrane-bound enzymes in the microsomal fractions used for the incubations *in vitro* and no contribution of other enzymes (e.g., cytosolic NQO1 or phase II biotransformation enzymes) that could contribute to BaP metabolism *in vivo* leading to different profile and concentrations of BaP reactive metabolites in rat livers *in vivo* and incubations with hepatic microsomes *in vitro*. Moreover, as BaP was administrated to rats by gavage, its transformation *in vivo* was probably already started in the gastrointestinal tract where the BaP-derived DNA adduct formation was not analysed. Total levels of BaP-DNA-adducts (dG-*N*^2^-BPDE and 9-hydroxy-BaP-4,5-epoxide-derived adduct) produced in incubations of BaP with hepatic microsomes isolated from all treatment groups *in vitro* correlated well with CYP1A1 enzyme activity determined by measuring the oxidation of Sudan I in these microsomes (*p* < 0.01; Figure 6B). *In vitro* adduct formation also correlated with MROD (CYP1A2 activity; *p* < 0.01; Figure 6C) and EROD (CYP1A1 activity; *p* < 0.05; Figure 6A). According to these findings, CYP1A2 may contribute to the BaP activation in rats, although it is essentially not involved in BaP activation in humans [7,9]. These interspecies differences have also been observed by others [35,60].

The estimated association between the induction of BaP biotransformation enzymes and BaP-induced genotoxicity was examined by Western blotting followed by specific immunodetection and determination of relative gene expression by qPCR. Combined administration of BaP and Sudan I led to a greater increase in CYP1A1, CYP1A2 and CYP1B1 protein levels than application of BaP or Sudan I alone (Figure 5). For CYP1B1, the amount of protein (Figure 5C) corresponded to changes in *CYP1B1* gene expression (Table 1). Compared to the control (untreated) group of rats, CYP1B1 expression both at protein and gene level exhibited the smallest, although very significant, increases after exposure of rats to BaP alone and the highest increases when rats were exposed to BaP before Sudan I. For CYP1A1/2 expression, the results at protein and gene level did not fully match. The observed increase in both *CYP1A1* and *1A2* gene expression in the liver of rats exposed to Sudan I prior to BaP treatment was the lowest among all groups and did not reflect an increased amount of protein. Although the expression of all CYP isoforms of interest is controlled by the Ah receptor [21,61], the mechanism of induction of individual genes may be different [62,63,64]. Other signal transduction pathways or post-transcription factors such as mRNA stabilisation/destabilisation, protein stabilisation, or protein transport [65,66] may play a role, as demonstrated for *CYP1A2*, expression of which may be independent of the Ah receptor [67,68,69,70]. Moreover, protein and gene expression of another enzyme involved in the BaP activation, mEH, was also found to be slightly increased in the rat livers after exposure to BaP alone, Sudan I alone or both compounds in combinations (Figure 7; Table 2). The induction effect of BaP on the level of mEH has been described previously in mice [50], suggesting that enhanced levels of mEH may contribute to the increased production of BaP-7,8-dihydrodiol in *in vitro* microsomal incubations and subsequently lead to increased dG-*N*^2^-BPDE formation as observed *in vitro* using rat liver microsomes exposed to the test compounds compared to control (untreated) rats.

**Figure 9:**
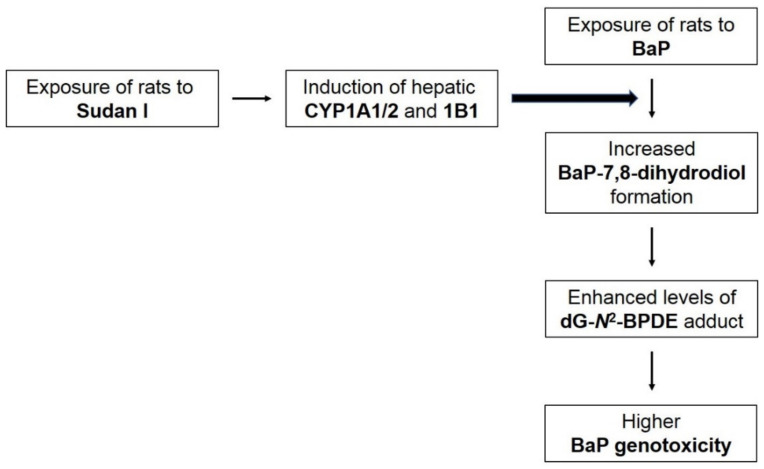
Proposed scheme of Sudan I impact on increased BaP-induced genotoxicity in rats exposed to Sudan I before BaP.

Our results emphasise the importance of investigating the effects of (geno)toxic compounds when acting together. Humans are usually exposed to complex mixtures of chemicals, which may contain several carcinogens whose effects on the body may be affected by their joint action. In the present study, we have shown that the industrially used dye Sudan I potentiates the genotoxicity of environmental pollutant BaP, and exposure to both chemicals at the same time may be dangerous to humans. According to our findings, Sudan I administrated to rats prior to BaP treatment induces the expression of cytochromes P450 1A1/2 and 1B1 in rat liver probably via the Ah receptor [71], which in turn contributes to increased BaP activation to the BaP-7,8-dihydrodiol, leading to higher dG-*N*^2^-BPDE DNA adduct levels (Figure 9). In addition, our results confirmed that in the rat, CYP1A1 is the BaP-activating enzyme that catalyses the conversion of BaP into reactive intermediates capable of forming DNA adducts. Finally, it is necessary to point out that there is the need for a comprehensive methodological approach when studying the mutual effect of compounds on the expression of biotransformation enzymes and on their individual carcinogenic properties. It might not be sufficient to focus only on the gene expression as changes at mRNA level may not necessarily correlate with protein expression and/or enzyme activity. Similarly, the study of metabolism for a given chemical is not sufficient to assess its genotoxic potential and DNA damage (e.g., DNA adducts) needs to be demonstrated as the level of genotoxicity is related to many other host factors including the tissue-specific induction of biotransformation enzymes, DNA repair or tissue distribution of the xenobiotics and their metabolites. The combination of *in vivo* and *in vitro* experiments can significantly contribute to a better understanding of genotoxic effects of carcinogenic compounds.

## 4. Materials and Methods

### 4.1. Chemicals and Enzymes

Glucose-6-phosphate, NADP^+^, NADPH, 7-ethoxyresorufin, 7-methoxyresorufin and BaP were obtained from Sigma-Aldrich (St. Louis, MO, USA), 5-bromo-4-chloro-3-indolyl-phosphate and nitro blue tetrazolium from Promega (Madison, WI, USA). Sudan I was purchased from BDH (Poole, UK), glucose-6-phosphate dehydrogenase from SERVA (Heidelberg, Germany) and Precision Plus Protein Western C Standard from Bio-Rad (Hercules, CA, USA). All other chemicals were of analytical purity or better. Supersomes™, microsomes isolated from insect cells transfected with a baculovirus construct containing cDNA of recombinant rat CYP1A1, 1A2 or human CYP1B1 and NADPH:CYP reductase, were purchased from Gentest Corp. (Woburn, MI, USA). The antibodies came from the following sources: a goat anti-rat CYP1A1 primary antibody from antibodies-online (Aachen, Germany), a rabbit anti-rat GAPDH primary antibody, an anti-chicken IgY- and an anti-rabbit IgG alkaline phosphatase secondary antibodies from Sigma-Aldrich (St. Louis, MO, USA), a rabbit anti-rat CYP1B1 primary antibody and anti-goat IgG alkaline phosphatase secondary antibody from Santa Cruz Biotechnology (Dallas, TX, USA), a rabbit anti-rat mEH primary antibody from MyBioSource (San Diego, CA, USA) and a chicken anti-rat CYP1A2 was prepared in the lab of Professor P. Hodek (Department of Biochemistry, Charles University, Prague, Czech Republic) as described previously [37]. Chemistry for RNA isolation, reverse transcription and qPCR was purchased as follows: GENEzol Reagent from Geneaid (New Taipei City, Taiwan), High-Capacity cDNA Reverse Transcription Kit with RNase Inhibitor, TaqMan Gene Expression Master Mix and TaqMan Gene Expression Assays for *CYP1A1*, *CYP1A2*, *CYP1B1*, *mEH* and *β-actin* from Thermo Fisher Scientific (Waltham, MA, USA).

### 4.2. Treatment of Rats

All animal experiments were conducted in accordance with the Regulations for the Care and Use of Laboratory Animals (311/1997, Ministry of Agriculture, Czech Republic), which is in compliance with the Declaration of Helsinki. Male Wistar rats (150 g, AnLab, Prague, Czech Republic), were housed in groups of three in wire cages at 22 °C with a 12 h light/dark period and ad libitum diet (ST-1 diet from Velaz, Prague, Czech Republic) and water access. For treatment, rats were randomly divided into five groups (*n* = 3/group) and treated according to the schedule in Table 3. Test compounds were all dissolved in sunflower oil. BaP was administered at a dose of 150 mg/kg body weight (bw) BaP, Sudan I at a dose of 50 mg/kg bw. All animals were sacrificed on day 3, 24 h after treatment at day 2. Liver tissues were collected, snap-frozen in liquid nitrogen and stored at ‒80 °C until further analysis.

### 4.3. BaP-DNA Adduct Detection by ^32^P-Postlabelling Analysis

Genomic DNA from liver tissue was isolated by a standard phenol–chloroform extraction method and DNA adducts were measured for each DNA sample using the nuclease P1 enrichment version of the thin-layer chromatography (TLC)–^32^P-postlabelling method as described previously [29]. After chromatography, TLC plates were scanned using a Packard Instant Imager (Downers Grove, IL, USA). DNA adduct levels were calculated as described [72]. Results were expressed as relative adduct labelling (RAL).

### 4.4. Preparation of Microsomes

Hepatic microsomes from all groups of rats were isolated as described previously [73]. Microsomes were isolated from 3 pooled livers of rats of each treatment group. Protein concentrations in the microsomal fractions were assessed using the bicinchoninic acid protein assay with bovine serum albumin as a standard according to the Microplate BCA Protein Assay Kit (Thermo Scientific, Waltham, MA, USA) protocol. The concentration of CYP was estimated according to Omura and Sato by measuring the absorption of the complex of reduced CYP with carbon monoxide [74].

### 4.5. Microsomal Incubations for BaP-DNA Adduct Formation

Incubation mixtures consisted of 50 mM potassium phosphate buffer (pH 7.4), 1 mM NADPH, pooled hepatic microsomal fraction (0.5 mg/mL protein) from all treatment groups, 0.1 mM BaP (dissolved in 7.5 μL dimethyl sulfoxide (DMSO)) and calf thymus DNA (0.5 mg) in a final volume of 750 μL. Incubations were carried out at 37 °C for 90 min [29]. Control incubations were carried out: (*i*) without microsomes; (*ii*) without NADPH; (*iii*) without DNA; and (*iv*) without BaP. After incubation, DNA was isolated by a standard phenol–chloroform extraction method. BaP-DNA adduct formation was determined by ^32^P-postlabelling as described above.

### 4.6. Western Blot Analysis

For the detection of individual CYP enzymes and mEH, 75 and 50 μg of microsomal proteins, respectively, were separated via sodium dodecyl sulphate polyacrylamide gel electrophoresis (10% acrylamide, Bio-Rad, Hercules, CA, USA). The polyvinylidene fluoride (PVDF) membrane after the electrotransfer was blocked in a solution of 5% skim milk in PBS-Triton X100 buffer (0.134 M NaCl, 1 mM NaH_2_PO_4_, 1.8 mM Na_2_HPO_4_, 0.3% (*w/v*) Triton X-100, pH 7.2) for 1 h at room temperature. CYP1A1 was detected with a goat anti-rat CYP1A1 primary antibody at dilution 1:1250, CYP1A2 was detected with a chicken anti-rat CYP1A2 primary antibody at dilution 1:1000, CYP1B1 was detected with a rabbit anti-rat CYP1B1 primary antibody at dilution 1:200 and mEH was detected with a rabbit anti-rat mEH primary antibody at dilution 1:1000 in 5% skim milk in PBS-Triton X100 buffer over night at 4 °C. After washing 3 times in PBS-Triton X100 buffer, membrane was incubated with alkaline phosphatase-conjugated secondary antibody anti-rabbit IgG (dilution 1:10,000), anti-chicken IgY (dilution 1:1000) and anti-goat IgG (dilution 1:5000) in 5% skim milk in PBS-Triton X100 buffer for 1 h at room temperature. Protein bands were visualised with the alkaline phosphatase substrate, 5-bromo-4-chloro-3-indolyl phosphate/nitro blue tetrazolium solution in ALP buffer (100 mM Tris/HCl, 150 mM NaCl, 1 mM MgCl_2_, pH 9). To assure comparable protein amount and expression, we routinely use GAPDH for normalisation of the Western blot data (dilution of the rabbit primary antibody was 1:5000).

### 4.7. Enzyme Activity Assays

The rat hepatic microsomal fractions were characterised for CYP1A1 enzyme activity using Sudan I hydroxylation [18,37] and 7-ethoxyresorufin *O*-deethylation (EROD) [36]. CYP1A2 enzyme activity was measured by 7-methoxyresorufin *O*-demethylation (MROD) [36].

### 4.8. Relative Gene Expression Analysis in Rat Livers

Total RNA was isolated from frozen livers of all rat groups and mRNA quantified by qPCR as described [75] with minor changes in chemicals used (see Section 4.1). The results and statistical significance were evaluated by the REST 2009 software (Qiagen, Hilden, Germany).

### 4.9. Microsomal Incubations for BaP Metabolites Detection

Incubation mixtures consisted of 100 mM potassium phosphate buffer (pH 7.4), NADPH-regeneration system (1 mM NADP^+^, 1 mM MgCl_2_, 10 mM glucose-6-phosphate, 1 U/mL glucose-6-phosphate dehydrogenase), pooled hepatic microsomal fraction (0.5 mg protein/mL) from all treatment groups, 0.05 mM BaP (dissolved in 5 μL DMSO) in a final volume of 500 μL. Incubations were started by adding the NADPH-regeneration system and carried out at 37 °C while shaking for 20 min. Control incubations were carried out without microsomes or without NADPH. After incubation, the reaction was stopped with 1 mL of ethyl acetate, followed by vigorous shaking. Next, 5 μL of 1 mM phenacetin in methanol (internal standard) was added. Individual samples were extracted twice with ethyl acetate by vigorous shaking for 2 min. Thorough separation of the two phases was performed by centrifugation at 13,000× *g* for 5 min. The upper phase (750 μL) was collected after each extraction and evaporated to dryness in a vacuum evaporator. Subsequently, the dried samples were stored at −20 °C until analysis by HPLC [9].

### 4.10. Statistics

For statistical data analysis we used Student’s *t*-test. All *p*-values are two-tailed and considered significant at the 0.05 level.

## 5. Conclusions

Exposure to Sudan I before BaP treatment resulted in the increased BaP-induced genotoxicity in rats *in vivo*, which seems to be mediated by the observed induction of hepatic CYP1A1/2 and 1B1 by Sudan I. The conjoint induction potential of BaP and Sudan I on the expression of CYP1A1 and CYP1B1 might influence their own biotransformation and affect their carcinogenic potential. Furthermore, increased CYP1A1/2 and 1B1 expression by the widespread contaminants tested in the present study can impact on an individuals’ susceptibility in humans to other environmental carcinogens or to used drugs by modifying their detoxification and/or activation pathways.

## Figures and Tables

**Table 1:** Relative mRNA expression of *CYP1A1*, *CYP1A2* and *CYP1B1* in livers from control (untreated) rats and rats exposed to BaP alone, Sudan I alone or Sudan I before BaP treatment (SI + BaP) or Sudan I after BaP treatment (BaP + SI).

mRNA	*CYP1A1*		*CYP1A2*		*CYP1B1*	
	Δc_T_ ± S.D.	Fold Change (to Control/BaP)	Δc_T_ ± S.D.	Fold Change (to Control/BaP)	Δc_T_ ± S.D.	Fold Change (to Control/BaP)
control	7.49 ± 0.122	-	−0.17 ± 0.007	-	11.36 ± 0.691	-
BaP	−4.06 ± 0.183	2990 ***	−4.96 ± 0.175	27.7 ***	−0.42 ± 0.071	3520 ***
Sudan I	−3.22 ± 0.526	1680 ***	−4.66 ± 0.461	22.5 ***	−1.76 ± 0.286	8860 ***
SI + BaP	−3.07 ± 0.187	1510 ***/0.5 ^#^	−4.45 ± 0.083	19.5 */0.7 ^#^	−1.40 ± 0.161	6920 ***/2.0 ^#^
BaP + SI	−4.25 ± 0.163	3420 ***/1.1	−4.89 ± 0.106	26.4 */1.0	−2.06 ± 0.109	10,900 ***/3.1 ^#^

Δc_T_ values relative to *β-actin* are means ± S.D. of three cDNA samples from independent RNA isolations from pooled livers of rats of one treatment group. Statistical significance was evaluated using the REST 2009 program: * *p* < 0.05, *** *p* < 0.001—results significantly different from control (untreated) rats; ^#^ *p* < 0.05—significantly different from rats treated with BaP alone.

**Table 2:** Relative expression of mEH mRNA in livers from control (untreated) rats and rats exposed to BaP alone, Sudan I alone or Sudan I before BaP treatment (SI + BaP) or Sudan I after BaP treatment (BaP + SI).

mRNA	*mEH*	
	Δc_T_ ± S.D.	Fold Change (to Control/BaP)
control	−1.97 ± 0.244	-
BaP	−2.65 ± 0.361	1.6 *
Sudan I	−3.04 ± 0.406	2.1 ***
SI + BaP	−2.58 ± 0.379	1.5 */0.9
BaP + SI	−3.26 ± 0.286	2.5/1.6

Δc_T_ values relative to *β-actin* are means ± S.D. of three cDNA samples from independent RNA isolations from pooled livers of rats of one treatment group. Statistical significance was evaluated using the REST 2009 program: * *p* < 0.05, *** *p* < 0.001—results significantly different from control (untreated) rats.

**Table 3:** Scheme of substance application to Wistar rats and application route. Sunflower oil was used as a vehicle. Dosage of the compounds: 150 mg/kg bw BaP or 50 mg/kg bw Sudan I.

Group Name	Treatment	Day 1	Day 2
control	untreated	Sunflower oil *(i.p.)*	Sunflower oil *(gavage)*
BaP	BaP	Sunflower oil *(i.p.)*	BaP *(gavage)*
Sudan I	Sudan I	Sunflower oil *(gavage)*	Sudan I *(i.p.)*
SI + BaP	Sudan I + BaP	Sudan I *(i.p.)*	BaP *(gavage)*
BaP + SI	BaP + Sudan I	BaP *(gavage)*	Sudan I *(i.p.)*

*i.p.*—intraperitoneal injection.

## Abbreviations

BaP	benzo[*a*]pyrene
BPDE	benzo[*a*]pyrene-7,8-dihydrodiol-9,10-epoxide
bw	body weight
CYP	cytochrome P450
DMSO	dimethyl sulfoxide
dG-*N*^2^-BPDE	10-(deoxyguanosin-*N*^2^-yl)-7,8,9-trihydroxy-7,8,9,10-tetrahydrobenzo[*a*]pyrene
EROD	7-ethoxyresorufin *O*-deethylation
GAPDH	glyceraldehyde 3-phosphate dehydrogenase
*i.p.*	intraperitoneal injection
mEH	microsomal epoxide hydrolase
MROD	7-methoxyresorufin *O*-demethylation
r	correlation coefficient
SI	Sudan I
TLC	thin layer chromatography
